# Pharmacokinetics comparison of two pegylated interferon alfa formulations in healthy volunteers

**DOI:** 10.1186/s40360-017-0192-z

**Published:** 2018-01-04

**Authors:** Marisa Boff Costa, Paulo Dornelles Picon, Guilherme Becker Sander, Hugo Nodarse Cuni, Carmen Valenzuela Silva, Rolando Páez Meireles, Ana Carolina Magalhães Andrade Góes, Nadia Maria Batoreu, Maria de Lourdes de Sousa Maia, Elizabeth Maciel Albuquerque, Denise Cristina de Souza Matos, Pedro Lopez Saura

**Affiliations:** 1 Center of Clinical Research, Hospital de Clínicas de Porto Alegre (HCPA), Universidade Federal do Rio Grande do Sul (UFRGS), Porto Alegre, RS Brazil; 2 Professor of Internal Medicine, Faculdade de Medicina do Rio Grande do Sul (UFRGS), Porto Alegre, RS Brazil; 3 Clinical Trials Division, Center for Biological Research, Havana, Cuba; 4 Clinical advice of BioManguinhos/Fiocruz, Rio de Janeiro, Brazil; 5 Immune Technology Laboratory BioManguinhos/Fiocruz, Rio de Janeiro, Brazil

**Keywords:** Pharmacokinetics, Pegylated interferon-alfa, Phase I

## Abstract

**Background:**

Several countries have used pegylation technology to improve the pharmacokinetic properties of essential drugs. Recently, a novel interferon alfa-2b protein conjugated to four-branched 12 kDa polyethylene glycol molecules was developed jointly between Cuba and Brazil. The aim of this study was to compare the pharmacokinetic properties of BIP48 (pegylated interferon alfa-2b from Bio-Manguinhos/Fiocruz, Brazil) to those of PEGASYS® (commercially available pegylated interferon alfa-2a from Roche Pharmaceutical).

**Methods:**

This phase I, single-centre, randomized, double-blind crossover trial enrolled 31 healthy male volunteers aged 19 to 35 who were allocated to two stages, either side of a 5-week wash-out period, with each arm lasting 14 consecutive days after subcutaneous administration of 180 μg of one formulation or the other (study or comparator). The main outcome variable was serum pegylated interferon concentrations in 15 samples collected during the course of the study and tested using an enzyme immunoassay.

**Results:**

There were no differences between formulations in terms of magnitude or absorption parameters. Analysis of time parameters revealed that BIP48 remained in the body significantly longer than PEGASYS® (T_max_: 73 vs. 54 h [*p* = 0.0010]; MRT: 133 vs. 115 h [*p* = 0.0324]; ke: 0.011 vs. 0.013 h(−1) [*p* = 0.0153]; t_1/2_: 192 vs. 108 h [*p* = 0.0218]).

**Conclusion:**

BIP48 showed the expected pharmacokinetic profile for a pegylated product with a branched molecular structure. Compared to PEGASYS®, the magnitude absorption was similar, but time parameters were consistent with slower elimination. Further studies should be conducted to evaluate the clinical implications of these findings. A phase II-III repeated-dose clinical trial is ongoing to study these findings in patients with chronic hepatitis C virus infection.

**Trial registration:**

This study is registered on the ClinicalTrials.gov platform (accession number NCT01889849). This trial was retrospectively registered in June 2013.

## Background

The development of new and better drugs is a permanent challenge within the pharmaceutical industry. A frequently used alternative is the pharmaceutical modification of known drugs to improve their pharmacokinetic (PK) and pharmacodynamic (PD) properties through the use of pegylation technology [1]. Pegylated (PEG) products have the advantage of minimizing many of the limitations associated with conventional therapeutic proteins (e.g., short half-life, immunogenicity, poor stability). The result is an increase in the length of time that the drug remains in the body [2].

Particular improvement in clinical efficacy and tolerability has been seen after development of PEG formulations of interferon (IFN) alfa. There are two PEG IFN-alfa products registered by international regulatory agencies on the market [3, 4], while others are being developed in several countries, including Brazil [5] and Cuba [6].

The Institute of Technology in Immunobiologicals/Bio-Manguinhos/Fiocruz, recognized as one of the largest Brazilian public laboratories in the field of immunobiological development and production, has been providing non-PEG recombinant human IFN alfa-2b since 2006 to a specific program of the Ministry of Health, the Exceptional (High Cost) Medicines Program, which consumes a significant portion of the health budget. In 2004, this Institute signed a technology transfer agreement with the Center of Genetic Engineering and Biotechnology (CGEB) of Havana, Cuba, for the development and production of PEG proteins.

This collaboration allowed the Institute to obtain monoPEG candidates with more than 95% purity, appropriate thermal stability, and a lower susceptibility to degradation by proteases than the non-modified protein [6]. Preclinical studies with a formulation of IFN alfa-2b conjugated to two-branched 40 kDa PEG molecules showed increased bioavailability and a significantly longer half-life than non-PEG IFN in rabbits [7], further tested in a PK comparison phase I clinical trial using PEGASYS® in healthy human subjects [8].

From the positive experience achieved with the first candidate in CGEB, a similar evaluation was designed for the second one, distinguished by its unprecedented spatial conformation, consisting of a branched PEG molecule containing four chains (12 kDa each) and for being produced at Bio-Manguinhos. This new molecule, named BIP48, will represent the first biologic medicinal product developed entirely in Brazil and will help the national Unified Health System reduce its dependence on expensive imports of PEG-IFN from the international market for treatment of hepatitis C virus (HCV) infection, a disease with high incidence and prevalence in the country.

The purpose of this work was to report on the early clinical evaluation of the aforementioned novel PEG-IFN product. The trial was designed as a randomized, double-blind, crossover, phase I study to compare the PK parameters of two formulations of IFN alfa (2b and 2a) conjugated to polyethylene glycol of different molecular sizes (48 kDa and 40 kDa respectively) in healthy volunteers.

## Methods

### Study design

This was a randomized, double-blind, crossover phase I clinical trial designed to enrol healthy volunteers with the aim of comparing PK parameters of BIP48 (PEG IFN alfa-2b, 48 kDa, from Bio-Manguinhos/Fiocruz) to those of PEGASYS® (PEG IFN alfa-2a, 40 kDa, from Roche Pharmaceutical).

Randomization was planned to generate two subject groups with administration of the products in two stages, separated by a 5-week wash-out period. Volunteers were admitted to a hospital and remained under medical observation for the first 96 h of the study. They then returned to the facility for regular blood sampling up to a total duration of 336 h (14 days). Over this time course, 15 blood samples per subject were drawn in each stage (before and after crossover).

### Subject selection

The sample comprised healthy male volunteers who met the following initial criteria for consideration: no history of chronic diseases, good oral health as evaluated by a dentist, no evidence of psychological disorders as evaluated by a psychiatrist, no history of acute illness during the previous 30 days, no significant clinical symptoms or signs on physical examination, all laboratory tests within standard normal limits, all imaging tests or scans within normal limits, and seronegative for human immunodeficiency virus and hepatitis B and C viruses.

The inclusion criteria were as follows: volunteers who signed an informed consent form; met the diagnostic criteria listed above; were male; aged 18 to 35 years; with a body mass index of 20 to 25.9; had no history of hypersensitivity to IFN alfa, to products derived from *Escherichia coli*, to PEG, or to any of the salts contained in the preparation; had never been treated with any type of IFN at any point prior to the test; were free from any history of chronic diseases such as autoimmune diseases, liver failure, decompensated cirrhosis, heart disease, kidney failure, diabetes mellitus, thyroid disorders, haemoglobinopathies, cytopaenia, psychiatric disease, retinopathy, or optic neuritis; had been free from acute infectious diseases for the previous 30 days; were not taking medications that modify immunity; had no drug allergies; no surgical interventions during the 6 months prior to the start of the study; had not donated blood; were not alcoholic or currently using alcohol; had not used illicit drugs in the last 6 months; and had not participated in any clinical trial involving a therapeutic intervention during the year prior to inclusion in this trial. Any of the items listed above was considered a criterion for exclusion.

### Treatment

Interventions: The first group of volunteers was randomized to receive 180 μg of PEGASYS® in the first stage, were followed for 14 days and then had a 5-week wash-out period. In the second stage, these same individuals received 180 μg of BIP48 and were followed for 14 consecutive days (AB sequence). The reverse drug sequence (BA) was administered in the remaining volunteers. Both PEG-IFN products were administered subcutaneously in the deltoid region after a 12-h fast.

### Allocation method

Randomization was performed in a single block, using the *R* software program. Volunteers were randomized into two groups of 16. At the time of enrolment, each participant was assigned a consecutive number.

### Study blinding

Since the product presentations of the two medicines were different, only the nurse who administered the injections knew which product was dispensed and administered to each volunteer.

### PK parameters of interest

The main outcome variable was the serum concentration of PEG IFN alfa, measured before administration and at 6, 12, 24, 36, 48, 60, 72, 84, 96, 120, 168, 216, 264, and 336 h after administration. Samples could be taken during an interval lasting from 5 min before to 5 min after the scheduled time, without breaking the protocol rules.

### Quantification of PEG IFN alfa in serum

A commercial enzyme-linked immunosorbent assay (ELISA) test was used to determine IFN-alfa levels. Assays were performed at the Immunobiology Laboratory (LATIM) at Bio-Manguinhos/Fiocruz. In this study, we used a specific kit for non-PEG IFN-alfa because there is no commercially available kit for quantification of PEG IFN.

### Correction factor for determination of serum PEG IFN alfa

To validate detection of PEG IFN using antibodies that recognize the conventional IFN-alfa molecule, we had to calculate a correction factor that would correlate PEG IFN with non-PEG IFN. Correction factors were determined for concentration values on the basis of the absorbance observed with the ELISA according to the type of molecule and serum dilution tested. This process was carried out in LATIM.

### Analysis of PK parameters

The PK profiles were defined by calculation of the main parameters that describe the process of drug absorption and elimination. The following PK parameters were calculated using experimental serum values for PEG IFN alfa, considering a non-compartmental PK model: maximum concentration (C_max_), area under the plasma concentration time curve (AUC); maximum time (T_max_); absorptiometry (CAV), half-life (t_1/2_), clearance (CL); distribution volume (VD); mean residence time (MRT); final elimination constant (Ke); mean absorption time (MAT); and duration of mean concentration value (HVD). All parameters were calculated in the Win-Nonlin Professional software environment, version 2.1 A (Pharsight Co., 1998).

### Statistics

#### Sample size

The number of subjects needed for this study was calculated on the basis of a similar comparative PK trial [8] in which a single 180-μg dose was administered to 16 healthy volunteers. Assuming a difference of up to 30%, loss to follow-up of 10%, alpha error of 0.10 and power of 80%, and using the results obtained for CAV_inf_, which is a PK parameter calculated from the ratio of two underlying parameters (C_max_ and AUC), we calculated that 29 to 32 subjects would have to be recruited.

#### Statistical analysis

For the PK measurements C_max_, AUC_(0-t)_ and T_max_, measures of central tendency and dispersion for the original parameters were estimated for each group (mean, median, 95% confidence interval for the mean, minimum and maximum values, standard deviation, and quartiles).

#### Ethics approval

The trial was conducted in compliance with the Declaration of Helsinki. The protocol was approved by the Ethics Committees of Hospital de Clinics de Porto Alegre (HCPA), the National Research Ethics Commission (CONEP), and by the Brazilian national regulatory authority (ANVISA).

#### Consent to participate

All volunteers provided written informed consent for participation in this study.

## Results

### Data set evaluated

We enrolled 31 healthy volunteers, 16 in the AB sequence and 15 in the BA sequence. All were considered in the demographic analysis (Table 1). The PK analyses were performed with results for 28 subjects, because there were three volunteers whose PEG IFN serum concentrations were not detected in most of the samples collected in the second stage (Subject #20, all assays except for 36 h; Subject #21, all assays except for 12, 24, and 36 h; Subject #31, all assays).

**Table 1:** Demographic and anthropological characteristics of study volunteers

Variables		Sequence AB (16)	Sequence BA (15)
Age	Mean ± SD	25 ± 5	27 ± 5
Weight	Mean ± SD	72 ± 6	69 ± 8
Height	Mean ± SD	174 ± 4	174 ± 6
Body mass index	Mean ± SD	23.6 ± 1.2	22.7 ± 2.0
Skin colour	White	14 (87.5%)	11 (73.3%)
	Black	2 (12.5%)	1 (6.7%
	Brown	0 (0%)	3 (20.0%)

### Demographic and baseline characteristics

The population included was predominantly white (80.6%), with a mean height of 1.74 m and mean body weight of 70.8 kg. Mean age was 26.1 years (range: 19 to 35).

### PK results

**Fig. 1:**
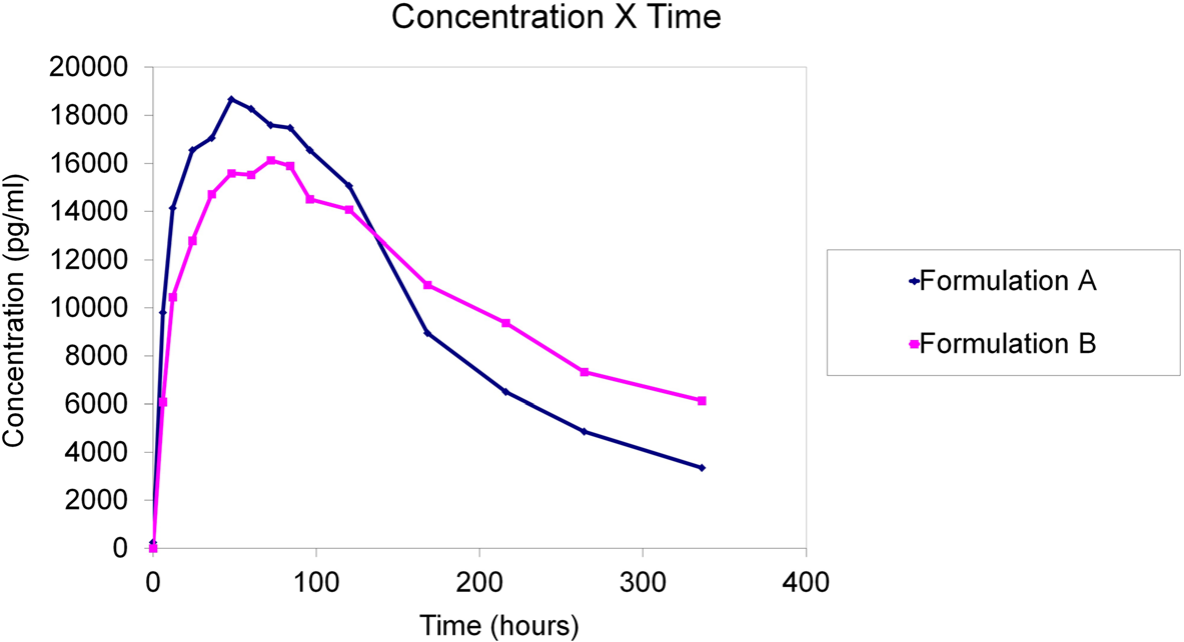
Mean serum concentration (pg/ml) X time (hours) curves for formulations A (PEGASYS®) and B (BIP48)

**Fig. 2:**
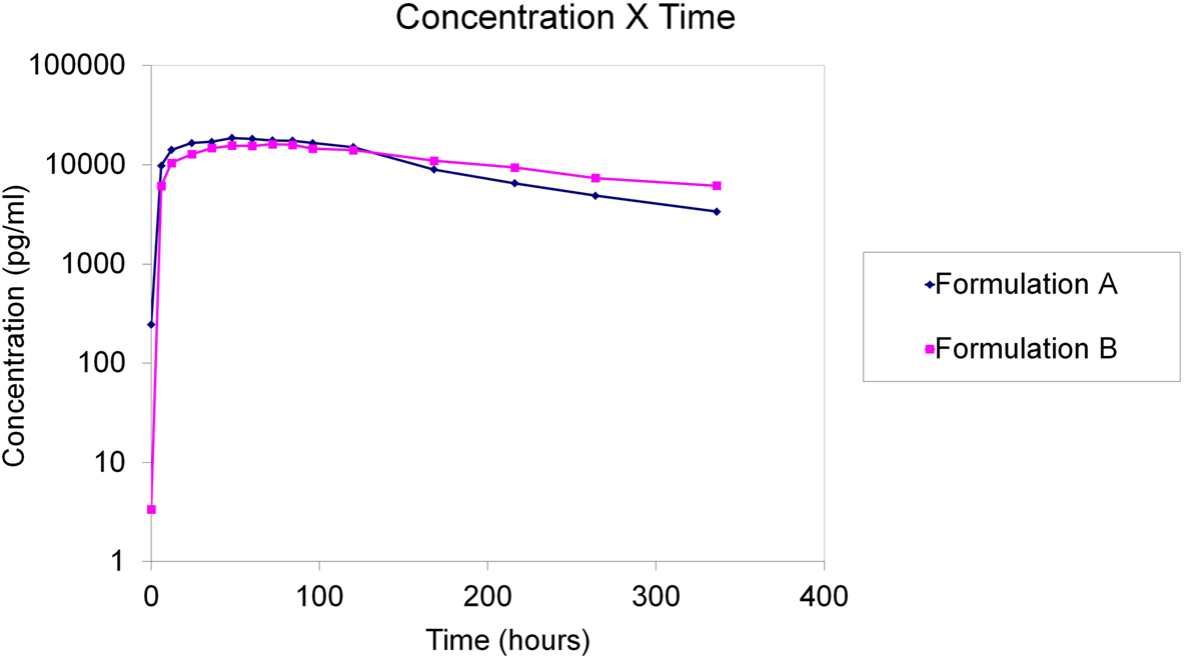
Mean logarithmic serum concentration (pg/ml) X time (hours) curves for formulations A (PEGASYS®) and B (BIP48)

Figures 1 and 2 shows PK profiles of serum PEG IFN alfa for both formulations. These plots show that both curves describe behaviour consistent with the corresponding PEG molecules. In both cases, the steps of absorption and elimination and the point of maximum concentration are well defined. Nevertheless, there are clearly visible differences along the PK profile, because PEGASYS® reached maximum serum concentration earlier and exhibited a faster rate of elimination from the body.

Table 2 lists measures of central tendency and dispersion for the PK parameters. The non-compartmental statistical model theory was also used and the results are shown in Table 3, also with measures of central tendency and dispersion for the PK parameters. These parameters support the results of the PK analysis conducted using the mathematical model.

**Table 2:** Means and standard deviations for pharmacokinetic parameters

Parameters		PEGASYS®	BIP48	*P*-Value (ANOVA)
T_max_ (hours)	Mean	54	73	0.001
	SD	18	22	
C_max_ (pg/ml)	Mean	19,960	18,630	0.3185
	SD	7066	8641	
Ke (^h-1^)	Mean	0.013	0.011	0.0153
	SD	0.007	0.013	
T ½ (hours)	Mean	107.8	191.8	0.0218
	SD	69.3	128.7	
AUC_(0–336)_ (pg/h/ml)	Mean	3,393,567	3,585,383	0.4649
	SD	1,562,761	2,197,201	
AUC_(0-inf)_ (pg/h/ml)	Mean	4,274,458	5,874,658	0.0053
	SD	2,373,083	4,066,639

**Table 3:** Noncompartmental analysis (Statistical Model) of pharmacokinetic measures for formulations A and B

Parameters		PEGASYS®	BIP48	*P*-Value (ANOVA)
MRT(_0–336_)	Mean	115	133	0.0324
(hours) CAV(_0–336_)	SD	27	37	
	Mean	0.006	0.007	0.3843
MRT (_0-inf_)	SD	0.002	0.004	
	Mean	177	300	0.0003
(hours)	SD	82	172	
CAV (_0-inf_)	Mean	0.005	0.005	0.2702
	SD	0.002	0.004	
MAT	Mean	28.1	30.9	0.2992
	SD	17	13.1	
HVD	Mean	161	216	0.3591
(hours)	SD	38	101	

BIP48 showed a longer duration of maximal concentration (73 vs. 54 h), longer residence time (MRT_(0–336)_ = 133 h vs. 115 h), longer duration of mean concentration (HVD = 216 h), longer half-life (t_1/2_ = 191 h), and shorter elimination constant (Ke = 0.008), all of which contributed to its having higher concentrations than PEGASYS® at the last evaluation time point, assuming that this difference is statistically significant to infinity. Absorption of the two products was similar according to CAV and MAT results. Analysis of variance only detected a “formulation” effect (difference between the two products) for T_max_, MRT_(0–336)_, Ke, t_1/2_, AUC_(0-inf)_, VD and MRT_(0 -inf)_.

In both stages of the study and in a high percentage of volunteers, AUC_(0–336)_ did not account for 80% of AUC_(0-inf)_. We therefore believe that the results for AUC_(0-inf)_ and MRT_(0-inf)_ should be disregarded for purposes of comparison, because they have a high component of dependence on the fitted model in the elimination phase. This was due to the large values obtained for the time parameters (T_max_, MRT_(0–336)_, t_1/2_, and Ke) and to the VD of BIP48.

## Discussion

This study was the initial step in clinical development of a Brazilian formulation of PEG IFN. The design was not a bioequivalence trial, since it was known a priori that the products had different molecular structures (48 kDa vs 40 kDa).

As required for any new biological therapeutic molecule, a phase I PK comparison study was performed on healthy volunteers. PEGASYS® was selected for use as comparator because both products have similarities in their branched molecular structures and a fixed dosage of 180 μg. The single dose administered was similar to others used in previous studies [8] and was deemed adequate, as it resulted in PEG IFN levels in serum easily detectable by ELISA even 336 h after product administration. In the present trial, a 5-week wash-out period was defined because PEGASYS® has a reference half-life of approximately 3 days [8], and the addition of another 2 weeks was estimated as enough time for total clearance from blood of the new PEG IFN product with a higher molecular weight.

The subject groups were comparable for all demographic and baseline variables, with no significant differences. Additionally, there was homogeneity between the groups in terms of baseline clinical and laboratory parameters before administration of PEG IFN alfa in both stages of the study.

The PEG IFN alfa products were administered to all volunteers at the planned and scheduled times. Clinical observations and sample collections were carefully timed and executed. The few deviations from the protocol that occurred at the time of sample collection had no impact on the results. There was no need to violate the randomisation list for any subject during the study.

PK analysis was performed using conventional parameters, which yield results that are easy to understand and compare with other data in the literature [9]. However, to facilitate analysis and decision-making in terms of the biological translation of a phenomenon to its relevant clinical expression, we added other variables, such as MAT and CAV, which can describe rate and extent of absorption more accurately [10].

Because this work represented the first clinical trial done with the PEG IFN 48 kDa formulation, a 14-day sampling period was planned on the basis of information reported for the reference product (PEGASYS®). This sampling time was insufficient to capture the full AUC. This was an unexpected finding, but does not constitute a limitation of the study. We believe that, when the crossover design was applied, with onset of the second stage of the study (after the wash-out period), a so-called residual effect occurred with impact on some PK parameters, including the average half-life. This means that, at the time of receiving administration of the product in the second stage, the individuals had not yet cleared the dose received in the first stage completely; therefore, the first dose made an unexpected contribution to serum concentrations, which were later detectable at the last time point of analysis (14 days). However, this did not affect the relationship between the study and comparator formulations, as demonstrated by the fact that, when stratifying the PK parameters by period, a tendency for a longer residence time in blood for BIP48 was maintained.

The PK profile curve shows that BIP48 has a prolonged duration time for its concentration in blood, as expected for a PEG IFN. The scientific value of this result is limited to providing insight into the process of absorption, distribution, and elimination of this specific product – an important premise that must be satisfied before it is approved for therapeutic use. These phase I data cannot be extrapolated to clinical efficacy [11]; this requires phase II exploratory efficacy trials.

However, the safety of the drug can be inferred from its PK profile, since a rapid increase in concentration to very high levels and a subsequent immediate fall (acute peak) is known to be related to toxicity rather than to efficacy. Our results show a more acute serum concentration curve for PEGASYS® than BIP48. This finding is directly related to the safety profile observed in healthy volunteers in this study, in which the control product caused a greater number of the typical flu-like adverse events of IFN-alfa. Nevertheless, no such data are presented herein, because they must be confirmed by the phase II trial.

The similarity between PK profile curves emphasizes the similarity of bioavailability between the study and comparator products. There were no differences in absorption fraction, mean absorption time, or transport from the site of administration to the circulatory system.

For products such as PEG IFN, which aim to obtain minimum effective levels for longer periods of time and thereby reduce the number of administrations, the most important PK parameters are those which indicate longer residence in the body, rather than those that reflect the magnitude of drug absorption [12]. Comparison of PK parameters showed that BIP48 offers clear advantages due to its considerably longer residence time in the body. These results can be explained by the differences in molecular weights and structures between the PEG IFN molecules [13].

The T_max_ obtained was higher for BIP48. However, in prolonged PK studies, such a difference of 48–72 h does not have clinical relevance. This parameter is of a discrete nature, and is dependent on fixed time and not on continuous determination throughout the sample evaluation period. Therefore, comparing it with other parameters that characterize absorption and elimination rates is of little value [9]. The slower elimination rate of BIP48, demonstrated by a Ke 2.3 times lower than that of PEGASYS®, in combination with its prolonged MRT and HVD, allows us to distinguish the kinetics obtained and can be explained by the chemical and spatial structures of the products [14].

The PK differences between the products were primarily due to the so-called formulation effect. Substantial variances between one study stage and another were observed in three volunteers who had undetectable serum levels after administration of BIP48. This result may be related to the idiosyncrasies of each individual, to absorption variability (differences at the injection site), intrinsic metabolic induction, varying levels of binding components (soluble circulating receptors, plasma proteins associated with the cell receptor), and/or variability in degradation speed or pattern.

The PK profile of any IFN or PEG IFN for parenteral administration should behave similarly between healthy volunteers and individuals with chronic HCV because, from a pharmacological standpoint, the intramuscular or subcutaneous routes of administration bypass the hepatic metabolic effect and guarantee the absorption of IFN into the bloodstream [15]. HCV does not cause any conditions that affect the renal elimination of IFN-alfa [16]. Furthermore, no PK interactions between ribavirin and IFN alfa-2b have been demonstrated when the two are given as combination therapy [17].

A phase II-III clinical study (NCT 01623336) with BIP48 in patients with chronic HCV, also compared to PEGASYS®, is ongoing. It is expected to characterize the PK profile of this product under a repeated dosing schedule and allow analysis of correlations between plasma levels of PEG IFN and relevant clinical response markers. In this new trial, the sampling time was prolonged with the addition of two observation time points (days 7 and 21) after the last administration of the product, in order to allow total characterization of the elimination phase and capture the full AUC. We expect to obtain the same superiority in time-related PK parameters achieved in the phase I trial reported herein.

The new direct-acting antivirals (DAA), given as part of IFN-free regimens, have demonstrated superior therapeutic efficacy and represent the immediate future in the treatment of chronic HCV infection [18]. However, virtually all countries in the Americas (including the United States) still recommend the use of PEG IFN in their national guidelines and include it in their essential drug formularies; the World Health Organization (WHO) kept it in its 19th Model List of Essential Medicines, published in April 2015, and recommends it as an alternative treatment for some patients, including those with liver cirrhosis and hepatitis C virus (HCV) genotype 3 infection and those infected by HCV genotypes 5 and 6 [19].

The therapeutic impact of DAA, although irrefutable, still has to be corroborated by long-term results. Recent research has attempted to describe the ability of these agents to induced prolonged relapse-free intervals as indicators of disease control (undetectable viral load) beyond 6 months after the end of treatment, which is the limit accepted to date as an efficacy criterion [20].

In addition, PEG IFN has other recognized therapeutic applications, such as in melanoma [21], chronic myeloid leukaemia [22], and renal carcinomas [23]. Therefore, the development of this new 48-kDa molecule that offers potential advantages in terms of efficacy and safety is very relevant.

## Conclusions

Our results demonstrate that BIP48 complies with international standards for medicinal products for human use. Its PK profile was characterized by a prolonged residence time as compared to an existing commercial formulation of PEG IFN. BIP48 is ready for evaluation in exploratory efficacy trials.

## Abbreviations

ANVISA: Brazilian national regulatory authority
ASCLIN: Clinical advisory team, bio-manguinhos/fiocruz
AUC: Area under the plasma concentration time curve
BIP48: Pegylated interferon alfa-2b
CAV: Absorptiometry
CGEB: Center of genetic engineering and biotechnology
CL: Clearance
Cmax: Maximum concentration
CONEP: National research ethics commission
DAA: Direct-acting antivirals
ELISA: Enzyme-linked immunosorbent assay
HCPA: Hospital de clinics de Porto Alegre
HCV: Hepatitis C virus
HVD: Duration of mean concentration value
IDMC: Independent data monitoring committee
IFN: Interferon
Ke: Final elimination constant
LATIM: Immunobiology Laboratory
MAT: Mean absorption time
MRT: Mean residence time
NUCLIMED: Center for clinical research on medications
PD: Pharmacodynamics
PEG: Pegylated
PEGASYS®: Pegylated interferon alfa-2a
PK: Pharmacokinetic
t1/2: Half-life
Tmax: Maximum time
UFRGS: Universidade Federal do Rio Grande do Sul
VD: Distribution volume
WHO: World health organization
